# Total Bio-Based Material for Drug Delivery and Iron Chelation to Fight Cancer through Antimicrobial Activity

**DOI:** 10.3390/nano13142036

**Published:** 2023-07-10

**Authors:** Vincenzo Patamia, Chiara Zagni, Roberto Fiorenza, Virginia Fuochi, Sandro Dattilo, Paolo Maria Riccobene, Pio Maria Furneri, Giuseppe Floresta, Antonio Rescifina

**Affiliations:** 1 Dipartimento di Scienze del Farmaco e della Salute, Università di Catania, Viale A. Doria 6, 95125 Catania, Italy; vincenzo.patamia@unict.it (V.P.); chiara.zagni@unict.it (C.Z.); 2 Dipartimento di Scienze Chimiche, Università di Catania, Viale A. Doria 6, 95125 Catania, Italy; roberto.fiorenza@unict.it; 3 Department of Biomedical and Biotechnological Sciences (Biometec), University of Catania, 95125 Catania, Italy; vfuochi@unict.it (V.F.);; 4 Center of Excellence for the Acceleration of Harm Reduction (Coehar), University of Catania, 95125 Catania, Italy; 5 IPCB-CNR, Via Paolo Gaifami 18, Institute for Polymers, Composites, and Biomaterials, Via Paolo Gaifami 18, 95126 Catania, Italy; sandro.dattilo@cnr.it (S.D.); paolomaria.riccobene@cnr.it (P.M.R.)

**Keywords:** resveratrol, curcumin, halloysite nanotubes, kojic acid, iron chelation, antibacterial

## Abstract

Bacterial involvement in cancer’s development, along with their impact on therapeutic interventions, has been increasingly recognized. This has prompted the development of novel strategies to disrupt essential biological processes in microbial cells. Among these approaches, metal-chelating agents have gained attention for their ability to hinder microbial metal metabolism and impede critical reactions. Nanotechnology has also contributed to the antibacterial field by offering various nanomaterials, including antimicrobial nanoparticles with potential therapeutic and drug-delivery applications. Halloysite nanotubes (HNTs) are naturally occurring tubular clay nanomaterials composed of aluminosilicate kaolin sheets rolled multiple times. The aluminum and siloxane groups on the surface of HNTs enable hydrogen bonding with biomaterials, making them versatile in various domains, such as environmental sciences, wastewater treatment, nanoelectronics, catalytic studies, and cosmetics. This study aimed to create an antibacterial material by combining the unique properties of halloysite nanotubes with the iron-chelating capability of kojic acid. A nucleophilic substitution reaction involving the hydroxyl groups on the nanotubes’ surface was employed to functionalize the material using kojic acid. The resulting material was characterized using infrared spectroscopy (IR), thermogravimetric analysis (TGA), energy-dispersive X-ray spectroscopy (EDX), and scanning electron microscopy (SEM), and its iron-chelating ability was assessed. Furthermore, the potential for drug loading—specifically, with resveratrol and curcumin—was evaluated through ultraviolet (UV) analysis. The antibacterial assay was evaluated following CLSI guidelines. The results suggested that the HNTs–kojic acid formulation had great antibacterial activity against all tested pathogens. The outcome of this work yielded a novel bio-based material with dual functionality as a drug carrier and an antimicrobial agent. This innovative approach holds promise for addressing challenges related to bacterial infections, antibiotic resistance, and the development of advanced therapeutic interventions.

## 1. Introduction

Millions of people die from cancer yearly, making it the world’s most prominent cause of death [1]. Researchers are dedicated to examining the origins of cancer and its progression, associated treatments, and postoperative interventions. Since growing evidence shows that bacteria can contribute to cancer’s formation and interfere with therapy by mediating its carcinogenesis and related infections, bacteria, which initially appeared to be independent of cancer, have attracted substantial interest among all cancer-related variables [2]. By triggering inflammatory responses and secreting bacterial enzymes, toxins, and oncogenic peptides, bacteria can make tumor growth worse. As bacteria may survive in malignant tissues due to their bacteria-friendly microenvironment and the severely compromised immune function of patients, cancer patients are more likely to acquire bacterial infections after therapy, even if it has been shown that bacteria have the potential to be exploited as anticancer agents [3,4]. Clostridium and salmonella have been shown to infect and survive within the human body, including in tumors. In fact, patients with solid tumors had a 42% Gram-positive infection rate and a 27% Gram-negative infection rate, compared to 47% and 30% for patients with hematological malignancies, respectively [5]. Moreover, surgery is frequently performed to remove most solid tumors, leaving scars or grafts at risk of infection, leading to inflammation, slow wound healing, and other consequences [6].

For example, the malignant tissue must be removed to treat skin cancer, but preventing infection and ensuring wound healing is challenging following surgery. Once an infection has set in, the delicate tissue will bleed, exude abundantly, cause discomfort, and cause fever, which can be exceedingly harmful to cancer patients. The malignant bone is often replaced with an orthopedic implant in cases of bone tumor resection. The probable infection, however, may cause insufficient soft tissue coverage, difficulties with the incision, and implant failure [2,7].

The need for antibacterial medications with novel or better modes of action is a health challenge of the greatest relevance in the age of rising antimicrobial resistance [8]. One of the main methods to ensure progress seems to be to increase or enhance the chelating properties of already-existing medications, or to discover new, nature-inspired chelating agents [9]. Resistance-based infections frequently do not respond to standard treatments, prolonging sickness, raising expenditures, and increasing the chance of mortality. Because the current antimicrobial medications either have too many adverse effects or tend to lose their efficacy due to the selection of resistant strains, the creation of innovative antimicrobial treatments is becoming more and more challenging [10].

These facts have led to several new techniques for impeding crucial biological processes in microbial cells. One such technique centers on using metal-chelating agents, which can disrupt the metabolism of the metals vital to the microorganism, hindering the uptake and bioavailability of those metals for critical reactions [11].

The biological function of metal-dependent proteins, such as metalloproteases and transcription factors, can be inhibited by chelation activity, which disturbs the homeostasis of microbial cells and blocks microbial nutrition, growth, and development, cellular differentiation, adhesion to biotic (such as extracellular matrix components, cells, and/or tissues) and abiotic (such as plastic, silicone, and acrylic) structures, and in vivo infection. Curiously, chelating drugs also increase the effectiveness of traditional antibacterial substances [6,11,12].

Nanomaterials have all of the characteristics needed to address these problems and create new technologies that can effectively target bacterial infections [13,14]; they also have the potential to be used to treat cancer itself [15]. First, nanoscale particles’ improved penetration and retention effects allow them to target tumor locations passively. Nanomaterials can be functionalized to actively target tumor tissues or cancer cells and accumulate at tumor sites through surface modification. For instance, cancer-targeting peptides can identify specific receptors [16,17,18,19], cationic elements can be added to nanomaterials’ surfaces to improve their tumor-penetrating ability [20], and nanomaterials’ shape or size can be altered to enhance tumor retention. Second, nanomaterials’ distinctive hydrophobic and hydrophilic architectures enable the loading of various medications in relatively high quantities, improving their solubility and safeguarding them against deterioration [21,22,23].

Natural clay nanotubes, known as halloysite, are one such nanoscale delivery method. It was discovered that halloysite is a practical and affordable nanoscale container for the encapsulation of physiologically active compounds, such as medicines and biocides [24,25]. The nearby alumina and silica layers and their water hydration provide a packing disorder that causes the layers to roll up and bend, forming multilayer tubes [26]. Compared to other nanotubes, using halloysite has several advantages. Its production is neither laborious nor dangerous, since it occurs naturally. Compared to other nanotubes (such as carbon nanotubes and inorganic nanotubes composed of tungsten, titanium, etc.), it is less costly [27,28]. Large particle size, an abundance of hydrophilic hydroxyl groups for functionalization, high stability in biological fluids, and inexpensive cost are all benefits of HNTs for drug delivery carrier applications [29,30]. Halloysite nanotubes are harmless up to concentrations of 75 mg/mL, and parallel laser confocal observation of fluorescently labeled halloysite absorption of cells revealed the material’s position inside the cells, close to the nucleus, demonstrating cellular uptake [30].

In this work, we modified HNTs with a derivative of kojic acid and then encapsulated them with resveratrol and curcumin—natural phenolic compounds with many beneficial effects on human health, such as antioxidant, anticancer, neuroprotective, and antiviral activities [31,32,33]. The successful preparation of this material was confirmed by various characterization methods. The novel material proved excellent drug-loading efficiency and chelating properties as proof of concept of a dual-acting antibacterial nanomaterial with resveratrol/curcumin and iron-depletion properties.

## 2. Materials and Methods

### 2.1. Materials

All of the required chemicals were purchased from Merck (Merck KGaA, Darmstadt, Germany) and used without further purification. The ^1^H- and ^13^C-NMR spectra were recorded at 300 K on a Varian UNITY Inova using DMSO-*d*_6_ as the solvent at 500 MHz for ^1^H-NMR and 125 MHz for ^13^C-NMR.

### 2.2. Synthesis of HNTs

#### 2.2.1. Synthesis of Chlorokojic Acid (**2**)

To a 100 mL round-bottomed reaction flask containing freshly distilled thionyl chloride (20 mL), kojic acid (**1**) (7.3 g) was added, and the mixture was magnetically stirred for 2 h. After one hour, a yellow-to-orange precipitate was formed. The product was collected by filtration, washed with petroleum ether, and then recrystallized from water to obtain colorless needles of chlorokojic acid (**2**) (5.2 g) at a 63% yield. The ^1^H and ^13^C NMR spectra of the compound were accordingly with the reported ones [34].

#### 2.2.2. Synthesis of HNTs/Kojic Acid Derivative

To a 10 mL round-bottomed reaction flask containing DMF (2 mL), we added halloysite (400 mg, 1 eq, 1.36 mmol) and triethylamine (1.14 mL, 6 eq, 8.16 mmol), and the mixture was magnetically stirred at room temperature for 30 min. Subsequently, chlorokojic acid (437 mg, 2 eq, 2.72 mmol) was added, and the reaction mixture was left to stir overnight at 80 °C. Then, the precipitate was collected by filtration, washed with acetone several times (5 × 10 mL), and placed in an oven at 65 °C overnight to obtain the final product (470 mg) at a 60% yield.

### 2.3. IR and UV–vis

FTIR analyses in the 4000–400 cm^−1^ region were conducted using an FTIR System 2000 (PerkinElmer, Waltham, MA, USA) with KBr as the medium. UV–vis spectroscopy (JASCO V-730 spectrophotometer, Easton, MD, USA) was used to determine the encapsulation efficiency (EE) and the drug-loading capacity (DLC).

### 2.4. Resveratrol and Curcumin Uptake

The loading of resveratrol on the HNTs–kojic acid system was carried out as described in the literature [30,35], at different weight ratios: 1:1, 1:2.5, 1:5, and 1:10 (resveratrol:HNTs–kojic acid *w*/*w*, Figure S1). Briefly, a resveratrol solution in water was prepared (7.5 mg L^−1^), and the correct amount of HNTs–kojic acid was suspended and kept under stirring for 1 h in the dark. Then, the suspension was centrifuged to separate the system from the uncharged drug. The loading capacities of curcumin were also evaluated, using different *w*/*w* ratios between the drug and the HNTs–kojic acid system. As described in the literature [36], a stock solution in ethanol was prepared (0.663 mg L^−1^), and curcumin was loaded on the HNTs–kojic acid system employing different weight ratios: 1:10, 1:50, 1:100, and 1:1000 (curcumin:HNTs–kojic acid *w*/*w*, Figure S2).

### 2.5. ICP/MS

Quantitative determination of iron ions in solution was performed by inductively coupled plasma mass spectrometry (ICP/MS) with a Nexion 300X (PerkinElmer Inc. Waltham, Massachusetts, USA.) instrument, using kinetic energy discrimination (KED) for interference suppression. Each determination was performed three times. The accuracy of the analytical procedure was confirmed by measuring a standard reference material—Nist 1640a trace element in natural water—without observing an appreciable difference. Batch equilibrium tests were carried out to calculate the metal ions’ removal percentage. In general, 10 mg of HNTs–kojic acid was immersed into iron(III) chloride (FeCl_3_) solutions (5 mL and pH = 6) at different initial Fe concentrations of 1.50 and 10.00 mg L^−1^. The vials were maintained under constant shaking at 25 °C and 180 rpm for 24 h, the suspension obtained was filtrated through a 0.22 nylon filter, and the solution was subjected to analysis by ICP-MS, as previously described.

### 2.6. SEM, EDX, and TGA

To study the morphology of the synthesized material, scanning electron microscopy (SEM) with a Phenomenex microscope was used. To increase conductivity before the test, the samples were pre-coated by gold sputter-coating. Images were then captured to examine the nanoclay morphology of the sample. The data were acquired and processed using Phenom Porometric 1.1.2.0 (Phenom-World BV, Eindhoven, the Netherlands). Energy-dispersive X-ray spectroscopy (EDX) was used to analyze the chemical elements in the material and determine its chemical composition. The material was subjected to thermogravimetric analysis (TGA) using a thermogravimetric apparatus (TA Instruments Q500) under a nitrogen atmosphere (flow rate: 60 mL/min) at a 10 °C/min heating rate, from 40 °C to 800 °C. The TGA sensitivity was 0.1 μg, with a weighting precision of ±0.01%.

### 2.7. Antibacterial Assay

The HNTs–kojic acid formulation was investigated for its antibacterial activity. *Escherichia coli* ATCC 25922, *Klebsiella pneumoniae* ATCC 700603, *Enterococcus faecalis* ATCC 29212, and *Staphylococcus aureus* ATCC 29213 strains were studied. The minimum inhibitory concentration (MIC) was determined through the broth microdilution technique, according to the recommendations stated in the Clinical and Laboratory Standards Institute (CLSI) document [37]. Briefly, a bacterial suspension of 0.5 McFarland was made for each strain under examination and, starting from the same suspension, the dilutions in broth were prepared to obtain a final concentration of 104 CFU/mL. The HNTs–kojic acid formulation was added at concentrations ranging from 1.5 mg to 24 mg. Each plate was prepared by including a positive control for bacterial growth (C+) and a negative control for sterility (C−). Each formulation was tested six times against each bacterial strain; the same experiment was repeated on a different day to ensure reproducibility.

## 3. Results and Discussion

### 3.1. Synthesis and Characterization

**Figure 1:**
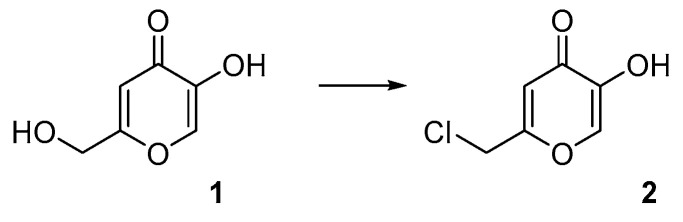
Synthesis of compound **2**.

**Figure 2:**
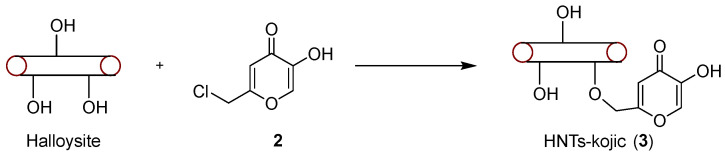
Synthesis of compound **3**.

To produce a derivative able to react with the hydroxylic groups of the HNTs, a derivative of kojic acid (**1**) was produced, giving chlorokojic acid (**2**) through a simple reaction with thionyl chloride (Figure 1). Compound **2** was then reacted with the HNTs, as reported in Figure 2, giving the final product **3**, named HNTs–kojic acid. TGA of **2**, HNTs, and **3** was performed, and the results are shown in Figures S3–S5. The maximal degradation rate of **2** is at 183 °C, while the highest-intensity peak of **3** is at 213 °C. In the DTG curve of **3**, a hump is visible at 194 °C, which is possibly due to chlorokojic acid, while the component of the peak at the higher temperature of 213 °C corresponds to the functionalized HNTs with chlorokojic acid. It is possible to infer that the percentage of functionalized HNTs with chlorokojic acid is higher than 10%.

Therefore, the material was characterized using IR, ICP/MS, SEM, and EDX, and the drug delivery capabilities were proven by drug-loading UV experiments with two natural molecules with antibacterial properties: resveratrol and curcumin [38,39,40].

**Figure 3:**
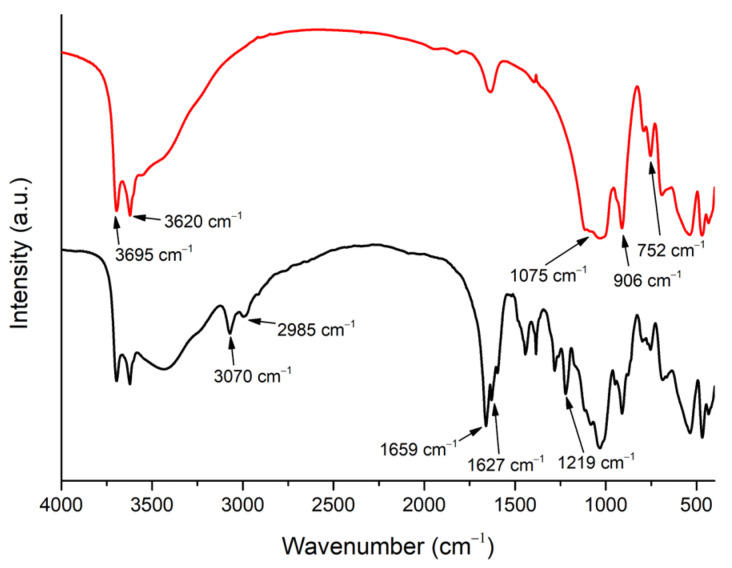
IR spectra of pristine halloysite (red line) and halloysite functionalized with kojic acid (black line).

The comparison of the IR spectra of HNTs and HNTs–kojic acid shows the successful functionalization of the halloysite nanotubes with kojic acid (Figure 3). In the spectrum of HNTs (red line), bands related to the OH groups are evident: the peak at 906 cm^−1^ is attributable to the Al-O-OH vibration, while the bands at 3695 and 3620 cm^−1^ can be attributed to the stretching vibration of the Al-OH groups. In addition, a strong peak related to O-Si-O is observed at around 1075 cm^−1^, and the peaks at 793 and 752 cm^−1^ can be assigned to the stretching mode of apical Si-O [35].

From the spectrum related to the functionalized HNTs–kojic acid (black line), we can see the presence of signals related to halloysite nanotubes, along with typical kojic acid signals: at 2985 and 3070 cm^−1^, the medium stretching of CH_2_ [41]; at 1659 cm^−1^, a strong signal related to conjugated ketone C=O; at 1627 cm^−1^, the typical C=C stretching of an unsaturated ketone [42,43]; and finally, the stretching associated with C-O at 1219 cm^−1^, which highlights the successful functionalization between the nanotubes and kojic acid.

ICP-MS spectra were recorded to verify the ability of the material to sequester iron(III) from the environment. The experiments revealed that HNTs–kojic acid chelates iron, i.e., eliminating gallium from solutions, with 65.33% retention of the ions when working with 1.50 mg/L of iron chloride, and 10.08% retention of the ions when working with 10.00 mg/L.

**Figure 4:**
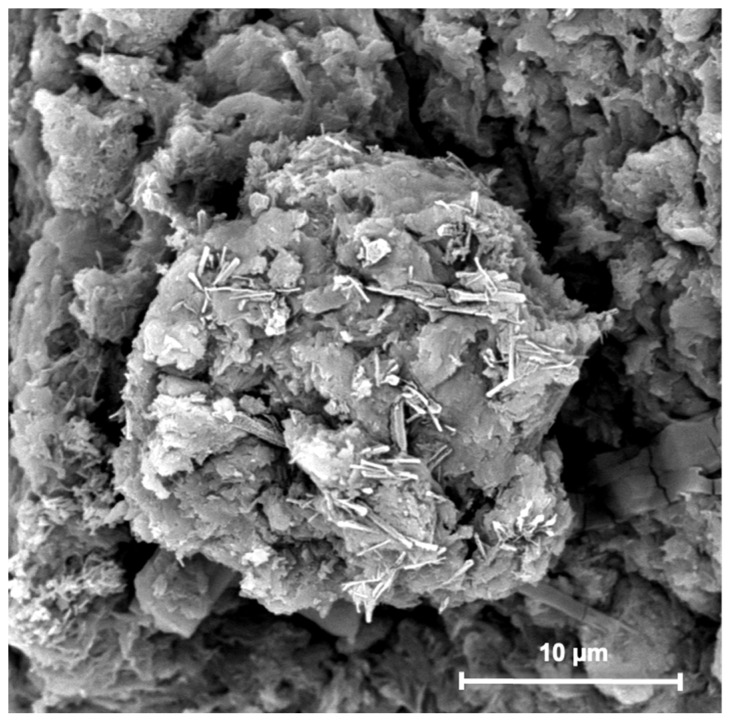
SEM image of HNTs–kojic acid.

Figure 4 shows SEM images obtained of HNTs–kojic acid spread on a flat support. The presence of pure and functionalized HNTs was verified by SEM, as most of the sample consisted of cylindrical tubes [44]. The average particle size calculated from the SEM images was 400 nm.

**Figure 5:**
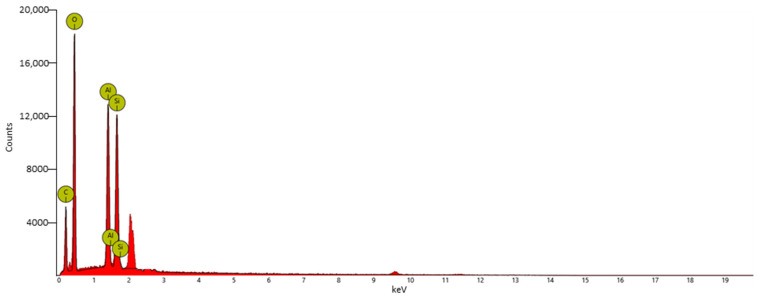
EDX spectrum of HNTs–kojic acid. The ordinate shows the counts, whereas the abscissa shows the keV. The unlabeled peaks at 2.1 and 9.6 keV correspond to gold, which is used to confer conductivity.

The SEM-EDX elemental mapping of surface-modified HNTs–kojic acid is shown in Figure 5 and Table 1. The major constituents of HNTs are oxygen, aluminum, and silicon. The HNTs–kojic acid shows the presence of carbon, oxygen, silicon, and aluminum.

### 3.2. Drug Loading and Release

Equations (1) and (2) describe the evaluation of drug-loading capacity (DLC) and encapsulation efficiency (EE) [35,45]:

(1) $$DLC=\frac{loaded\;drug\;amount}{total\;HNTs-kojic\;amount}$$

(2) $$EE=\frac{loaded\;drug\;amount}{total\;drug\;amount}$$

**Figure 6:**
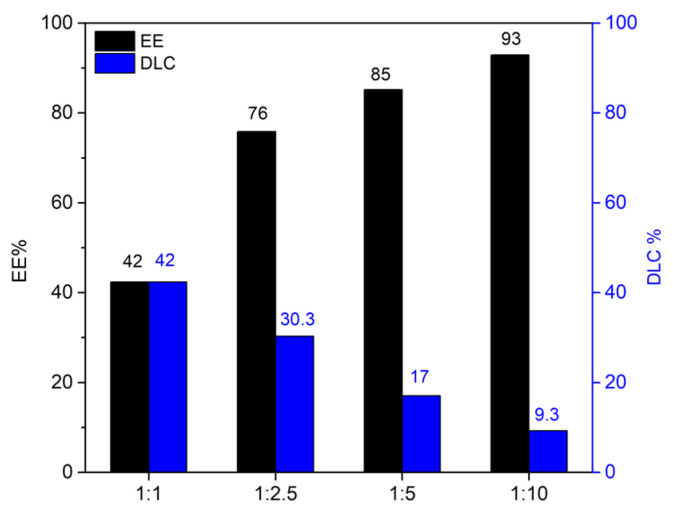
Encapsulation efficiency (EE) and drug-loading capacity (DLC) of resveratrol on the HNTs–kojic acid.

**Figure 7:**
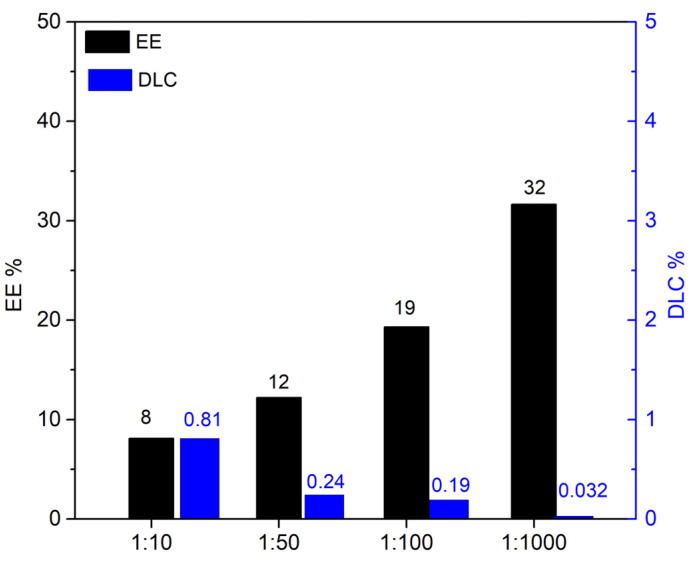
Encapsulation efficiency (EE) and drug-loading capacity (DLC) of curcumin on the HNTs–kojic acid.

From Figure 6, it is clear that it is possible to obtain the highest EE with the highest amount of material. The results (DLC at a 1:1 loading ratio) are consistent with the literature concerning the release of resveratrol [35]. The same experiments were conducted with curcumin; once again, the DLC and EE values (Figure 7) detected by UV analysis were in excellent agreement with the literature [36,46,47].

**Figure 8:**
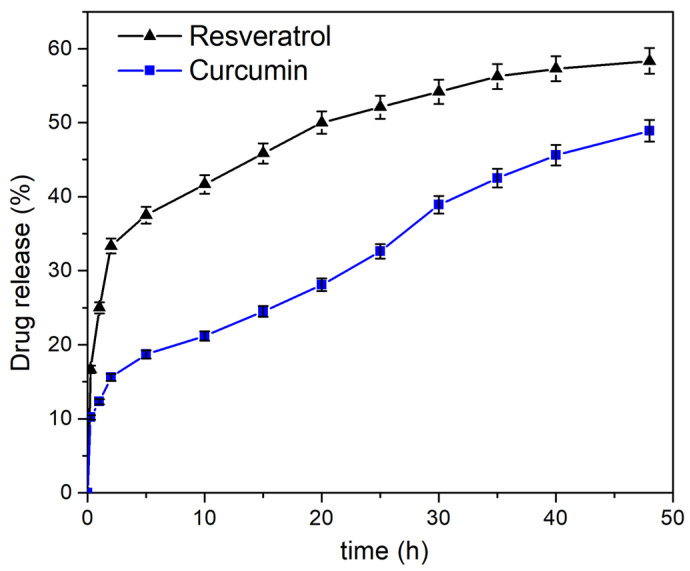
Release kinetics of resveratrol and curcumin from HNTs–kojic acid.

The difference between the loading capacity of resveratrol and curcumin with the HNTs–kojic acid system can be reasonably attributed, as reported in the literature, to the different solubility of the two drugs (resveratrol is weakly soluble in water, whereas curcumin is soluble in ethanol) [30,35,36,48]. However, the good interaction of the drugs with the peculiar nanotubular structure of the HNTs–kojic acid sample is promising for the possible application of this material in the field of drug carriers. The release kinetics of the drugs is reported in Figure 8. For these tests, we used the 1:1 resveratrol/HNTs–kojic acid and 1:100 curcumin/HNTs–kojic acid loading ratios to appreciate the drugs’ release better. Consistent with the literature [30,35,49,50], up to 10 h, the release kinetics was relatively fast (about 40% for the resveratrol and 20% for the curcumin), after which the release became slower, with a waiver of 50% and about 30% in 20 h for the resveratrol and the curcumin, respectively, to reach 60% for the resveratrol and the 45% for the curcumin at 40 h. The latter drug was characterized by a slower kinetics compared to resveratrol. The quick drug release in the first hours was due to the rapid dissolution of the drugs adsorbed in the nanotubes of the halloysite, while the other drug molecules were gradually released from the sample surface, and this delivery was also affected by the diffusion phenomena.

### 3.3. Microbiological Assays

**Figure 9:**
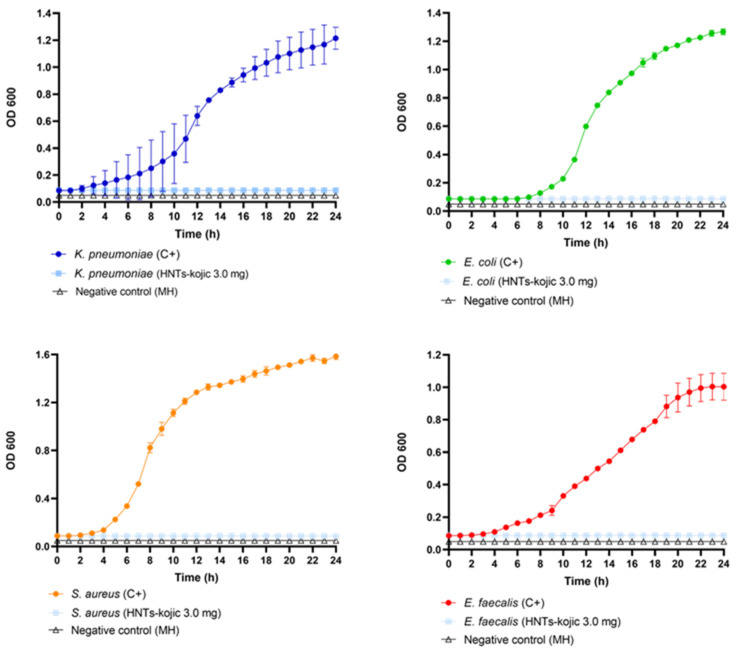
Growth curves (24 h) of the tested bacteria treated with the HNTs–kojic acid formulation.

The antibacterial assay was evaluated following CLSI guidelines. The results suggested that HNTs–kojic acid (**3**) had great antibacterial activity against all pathogens tested, with an MIC value equal to 3.0 mg, as shown in Figure 9. As evident from the bacterial growth curves, the new material demonstrated excellent antibacterial efficiency against both Gram-positive and Gram-negative strains.

## 4. Conclusions

The use of antibiotics in cancer therapy has advanced significantly, especially with the rapid development of nanomedicine. Significant efforts have been made to build nanosystems to improve the efficacy of medication delivery, adopt novel therapeutic agents or treatment modalities to reduce drug resistance, and implement local antimicrobial therapy. Nevertheless, there are always new difficulties associated with new approaches. Nanocarriers’ size and surface charge are crucial, because they can affect the detection, adsorption, and removal of nanoparticles, influencing how widely distributed they are throughout the body. Even after they have reached the tumor site, smaller-scale nanoparticles in the circulatory system can be quickly cleared and leaked back into the circulation, whereas larger nanoparticles have strong retention but are unable to penetrate cells.

Antibacterial nanosystems have made impressive advances in cancer therapy overall, but there is still more work to be done before they can be used effectively and safely [51,52]. Because of their high biocompatibility and on-demand drug delivery, HNTs have received much interest recently in the biomedical area; by adding the appropriate chemotherapy or antibacterial medications, HNTs could be employed for antitumor or antibacterial treatments, as well as for the treatment of other diseases [29].

This work proposed the synthesis of a new nanomaterial based on HNTs and kojic acid (HNTs–kojic acid). HNTs–kojic acid was characterized by several techniques, and its iron chelation and drug delivery capabilities were well documented.

The system was developed to serve as a proof of concept of a dual-acting antibacterial nanomaterial with resveratrol/curcumin and iron-depletion properties for cancer and other infection-related applications. The chelating moiety of maltol was chemically bonded to the material, and the resveratrol/curcumin was incorporated into the HNTs cavity.

This new material proved that applying dual action due to the drug and the iron-chelating properties is possible with HNTs. Moreover, the results of the antibacterial evaluation conducted on this new formulation are auspicious. It was observed that, even at low concentrations, it exhibited antibacterial activity against both Gram-positive and Gram-negative bacteria. This suggests that the iron chelation action of the nanotubes is exceptionally efficient.

These findings open the way for further research and applications of the developed material in cancer treatment and other infection-related issues. Even if the use of HNTs in the treatment of tumors is still in the experimental stage, this new prototype material could have a wide range of applications in cancer and other fields, e.g., using HNTs–kojic acid with the already studied molecules, i.e., resveratrol or curcumin; using HNTs–kojic acid as a drug carrier for other molecules (antibacterial or others); reevaluation/potentiation of old antibiotics; or combined use of the aforementioned solutions.

## Figures and Tables

**Table 1:** Atomic and weight concentrations of HNTs–kojic acid obtained by EDX analysis.

Element Symbol	Atomic Conc. %	Weight Conc. %
O	45.27	45.06
C	40.70	30.41
Al	6.76	11.35
Si	6.22	10.87

## Data Availability

Not applicable.

## Supplementary Materials

The following supporting information can be downloaded at: https://www.mdpi.com/article/10.3390/nano13142036/s1, Figure S1: UV spectra of the loading capacity of resveratrol on the HNTs–kojic acid system with different ratios. Figure S2: UV spectra of the loading capacity of curcumin on the HNTs–kojic acid system with different ratios. Figure S3: TGA of HNTs. Figure S4: TGA of **2**. Figure S5: TGA of **3**.
