# Supplementary material for: Total Bio-Based Material for Drug Delivery and Iron Chelation to Fight Cancer through Antimicrobial Activity
Source: Nanomaterials (Basel). 2023 Jul 10;13(14):2036. doi: 10.3390/nano13142036 (PMC10384306; doi:10.3390/nano13142036)
Supplement: Supplementary file 1 [file nanomaterials-13-02036-s001.zip › nanomaterials-2465074-supplementary.pdf]

*Supplementary Materials*

# Total Bio-Based Material for Drug Delivery and Iron Chelation to Fight Cancer through Antimicrobial Activity

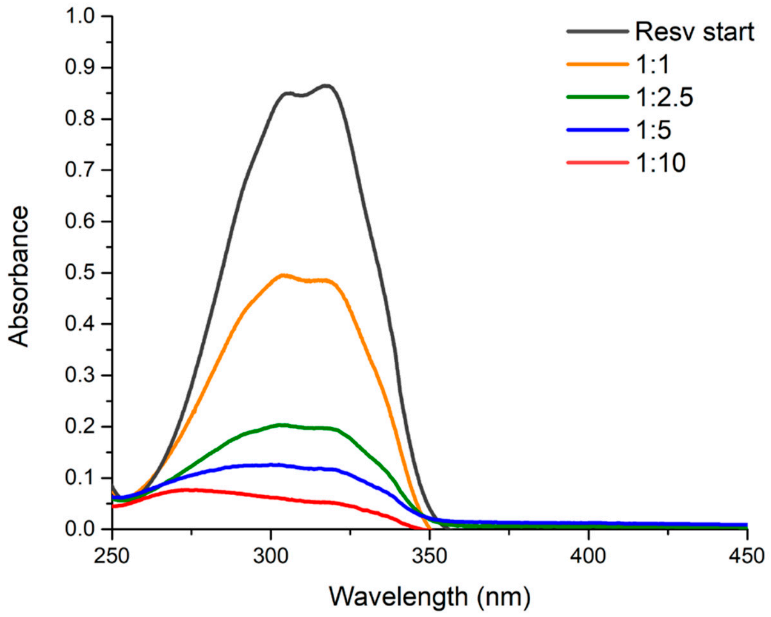


**Figure S1.** UV spectra of the loading capacity of resveratrol on the HNTs-kojic system with different ratios.

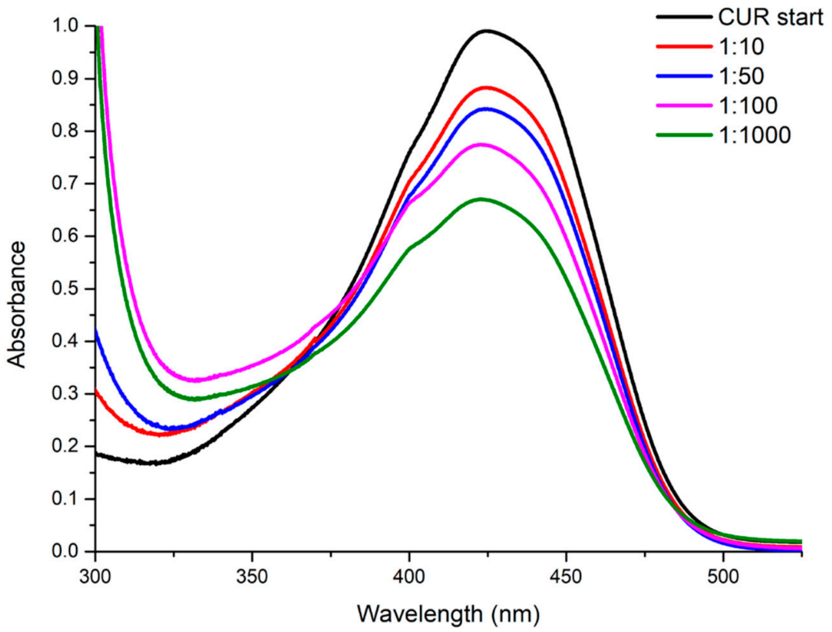


**Figure S2.** UV spectra of the loading capacity of curcumin on the HNTs-kojic system with different ratios.

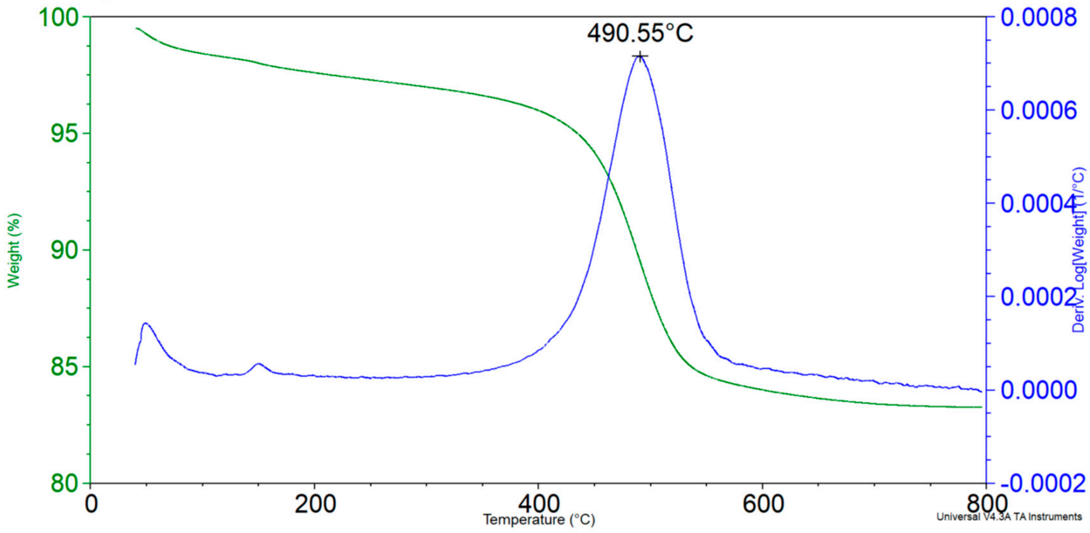


**Figure S3.** TGA of HNTs.

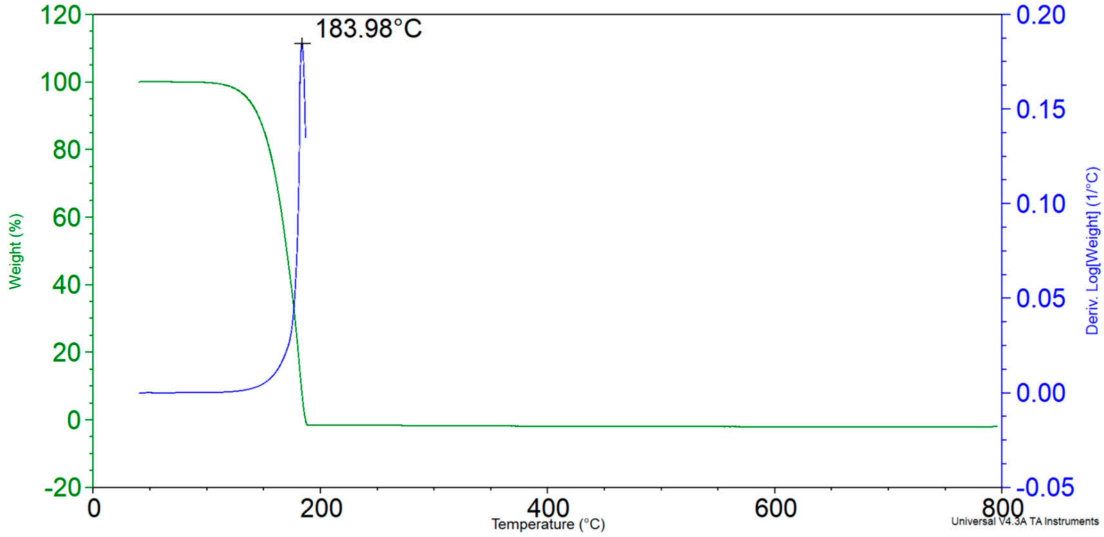


**Figure S4.** TGA of **2**.

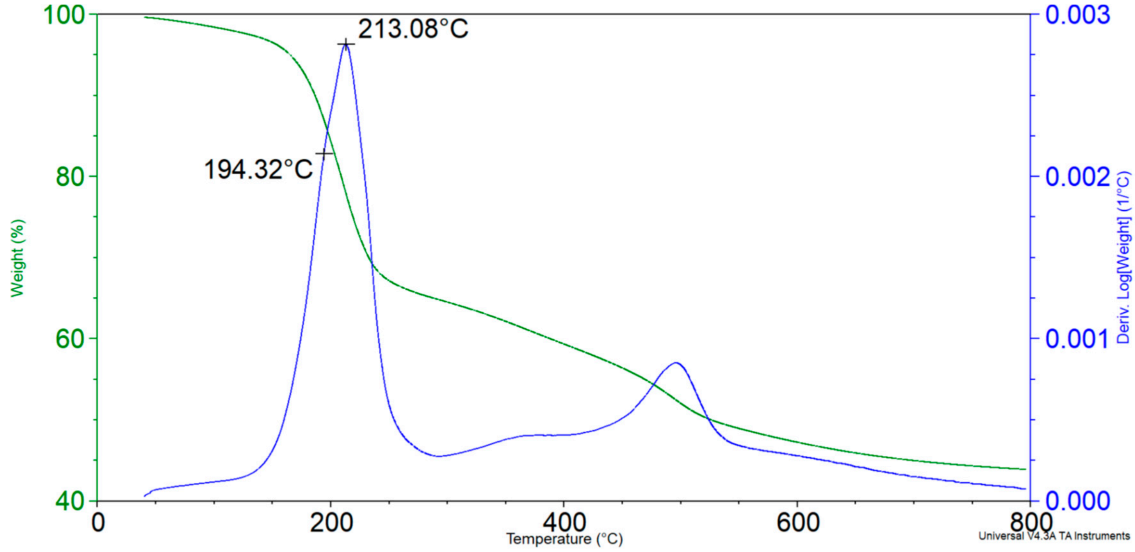


**Figure S5.** TGA of **3**.
